# Contemporary portable oxygen concentrators and diverse breathing behaviours -- a bench comparison

**DOI:** 10.1186/s12890-019-0980-x

**Published:** 2019-11-19

**Authors:** Dion C. Martin

**Affiliations:** ResMed Science Center, ResMed Ltd, Elizabeth Macarthur Drive, Bella Vista, Sydney, Australia

**Keywords:** Portable oxygen concentrator (POC), Nasal cannula, Long-term oxygen therapy (LTOT), Pulsed oxygen delivery, Nocturnal oxygen therapy (NOT), Oxygen-conserving technology, Oxygen use efficiency, Lung simulator, Nasal cannula, Chronic obstructive pulmonary disease (COPD)

## Abstract

**Background:**

Decades of clinical research into pulsed oxygen delivery has shown variable efficacy between users, and across a user’s behaviours (sleep, rest, activity). Modern portable oxygen concentrators (POCs) have been shown as effective as other oxygen delivery devices in many circumstances. However, there are concerns that they are not effective during sleep when the breathing is shallow, and at very high respiratory rates as during physical exertion. It can be challenging to examine the determinants of POC efficacy clinically due to the heterogeneity of lung function within oxygen users, the diversity of user behaviour, and measurement issues. Representative bench testing may help identify key determinants of pulsed-oxygen device efficacy.

**Methods:**

Three contemporary devices were bench-evaluated across three simulated breathing behaviours: activity, rest, & oronasal breathing during sleep. Emphasis was placed on breathing patterns representative of oxygen users.

**Results:**

All three POCs performed well during simulated breathing during exertion and at rest. Differences in triggering ability were noted for the scenario of oronasal breathing during sleep.

**Conclusions:**

The results are supportive of contemporary POC triggering abilities. The differences shown in ultimate trigger sensitivity may have relevance to oronasal breathing during sleep or other challenging scenarios for pulsed oxygen delivery, such as dominant mouth breathing during exertion or unfavourable nasal geometry.

## Background

For those with severe COPD prescribed long-term oxygen therapy, ambulatory oxygen can promote exercise tolerance and facilitate social interaction [1]. Given the choice, most subjects would prefer the lightest-weight system that provides effective oxygen therapy over a sufficient duration [2]. Efficient dispensing of oxygen may facilitate this.

**Fig. 1:**
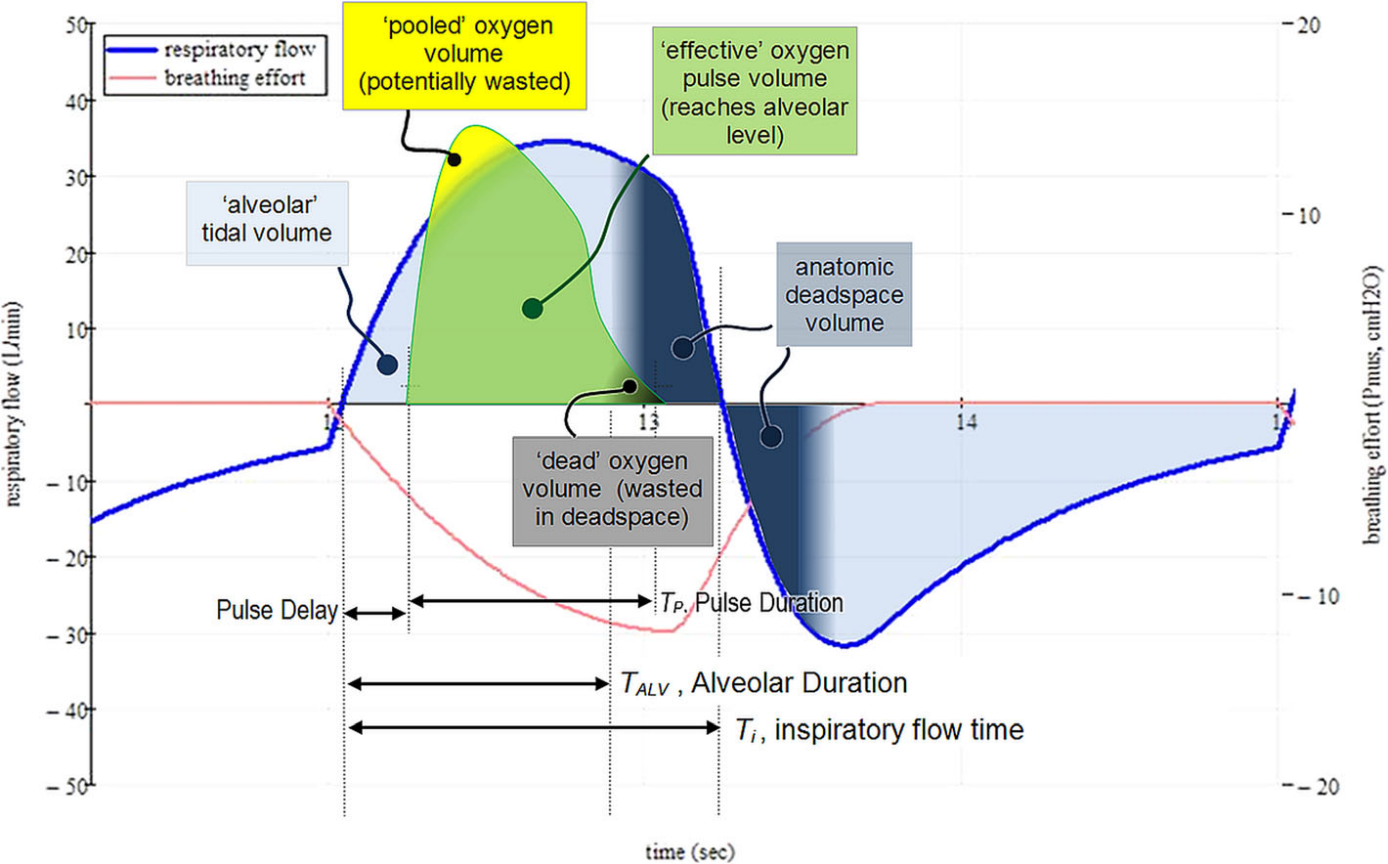
Respiratory flow and oxygen flow for a single breath during pulsed dose oxygen delivery. Oxygen is potentially ‘useful’ to the patient if delivered within the ‘alveolar’ tidal volume. Wastage of oxygen *may* occur if the oxygen pulse flow exceeds inspiratory flow, depending on the prevailing conditions. Note that the timing datum is the start of inspiratory flow

The traditional home oxygen therapy is low-flow oxygen, comprising a continuous oxygen flow delivered via nasal cannula. This method of delivery is simple but inherently wasteful. The oxygen delivered throughout expiration is wasted except for any which may ‘pool’ for subsequent inhalation. Also wasted is the oxygen flow during late inhalation, which reaches only the conduit airways rather than gas-exchanging lung units. Figure 1 shows (in dark blue) the portion of the inspiratory flow destined for anatomic dead space. If instead the oxygen is delivered only intermittently, at those times productive for gas exchange, oxygen is conserved. An oxygen source – be it lightweight compressed oxygen, liquid oxygen or a battery powered oxygen concentrator – can be combined with an oxygen conserving device which releases an oxygen pulse only when an inhalation is detected. Such devices are known as pulsed oxygen delivery systems (PODS) [3].

It has been demonstrated that specific PODS devices can be efficacious across a full range of breathing behaviours: at rest, during exercise, and during sleep [4, 5]. But pulsed oxygen technology is diverse, and not all studies are so positive. Given the heterogeneous nature of COPD this is to be expected; clinical studies of any oxygen therapy in these patients uniformly demonstrate a wide variability in oxygenation. But for pulsed oxygen systems in particular, device technical factors may potentially play a role. This was emphatically demonstrated by Palwai and colleagues in 2010, in a comprehensive clinical & technical investigation into one class of pulsed-oxygen devices: oxygen conservers [6]. The efficacy results were confronting: some devices worked better at rest than with exercise, but some worked poorly during both, and one performed no better than room air. Their data suggest a substantial triggering unreliability in a majority of their tested conserver devices. The authors deemed the consequences of the errant triggering to be highly clinically relevant, and suggested earlier published research had inadequately examined device performance, such as failing to verify pulse volumes or pulse synchrony.

Portable oxygen concentrator (POC) devices are a relatively recent development in pulsed oxygen delivery. These are gaining in popularity as technology evolves and benefits to the user are established, such as size, weight, operating duration, or their ability to be used on passenger flights. Like many PODS technologies, POCs deliver an oxygen pulse via nasal cannula to the user when inhalation is detected, sensed as a negative pressure fluctuation within the cannula. Their sophisticated triggering electronics *may* be more effective than that of the older oxygen conservers studied by Palwai et al. [6]. But the 2013 investigation of Leblanc et al. [7] into 3 different POCs demonstrated that rated oxygen output failed to correlate with oxygen saturation achieved during exertion. Their study did not investigate why, but pulse asynchrony may again be suspected.

It is understandable then that a recent expert review into home oxygen therapy relayed concerns that pulsed oxygen delivery by portable concentrators may not be effective during sleep or at high respiratory rates [2]. Investigating these concerns through clinical study is complicated by heterogeneity of oxygen users’ condition and behaviour, and by measurement difficulties. Representative bench testing may be useful in scrutinizing key determinants of pulsed-oxygen device efficacy. Bench testing of pulsed oxygen delivery systems is well established, but typically simulates the relatively unchallenging scenario of an awake adult COPD patient at rest: substantial tidal volumes, a well-fitted cannula, 100% nasal breathing, and sometimes with a span of respiratory rates [8–12].

The focus of our bench study was to explore the reliability POC triggering across a *broad* range of breathing behaviours which are common in COPD patients, including the identified areas of concern of shallow breathing and high respiratory rates [2]. In designing our tests, we emphasised the use of credible adult COPD breathing patterns to maximise clinical relevance, applied to 3 representative modern POCs.

## Methods

We simulated the adult COPD patient scenarios listed below. Details on the associated modelling rationale and breathing simulator settings are summarised in Table 1.

1. The onset of physical exertion with the associated dynamic increase in breath rate, spanning from 20/min to 34/min. This scenario assesses a POC’s ability to maintain synchrony during *changing and elevated breath rates*. Although a shift from nasal to oronasal breathing might be common during exertion, this test maintains 100% nasal breathing throughout given that trigger sensitivity is evaluated in the other scenarios.
2. A small adult patient at rest with 100% nasal breathing, as an example of *lower-than-typical tidal volume*.
3. An example of an adult with *substantially reduced nasal ventilation*, as represented by oronasal breathing during sleep.

**Table 1:** Modelling details for breathing sequences

Scenario and modelling guidance	Breath rate /min	Effort	Nasal Tidal Volume	R_in_ / R_ex_ cmH_2_O/ L/sec	C_rs_ mL/ cmH_2_O
COPD patient, onset of exertion: Progressive increase in breath rate, inspiratory effort amplitude, and expiratory effort contribution, guided by references [13, 14].	20–34	Figure 2(b)	234 mL–700 mL (100% nasal)	8 / 13	75
Low demand COPD patient at rest: Lower-than-typical volume for adult COPD. From Fig. 1 of reference [15], the lowest minute ventilation for an awake COPD patient in this cohort was 5.1L/min. A chronic stable COPD resting breath rate of 17/min was adopted [16].	17	Figure 2(a) dashed curve	304 mL (100% nasal)	6 / 11	75
COPD patient, reduced nasal fraction: Sleeping COPD patient with average minute ventilation, breathing through both nose and mouth. Oronasal breath partitioning guided by Fig. 4 of [17]: for their subjects over 45 years old, oral proportion was 51% (median) or 45% (mean). An oral proportion within this span was used: 47%. Median ventilation for a COPD patient during REM sleep: 5.9 L/min from Fig. 1 of reference [15]. Mean breath rate during REM for nocturnal desaturators, Fig. 3 of [15]: 17.6/min. Compliance reduced and resistance increased consistent with supine posture and sleep [18].	17.6	Figure 2(a) solid curve	182 mL (53% nasal, total V_T_ of 343 mL)	12/ 15	65

R_in_, inspiratory resistance, cmH_2_O/ L/sec; R_ex_, expiratory resistance, cmH_2_O/ L/sec; C_rs_, compliance of the respiratory system, mL/cmH_2_O; REM, rapid eye movement; V_T_, tidal volume

The devices tested were Inogen’s Inogen One G3 (Device A), ResMed’s Mobi (Device B), and Philips Respironics’ SimplyGo Mini (Device C), prepared according to their respective user instructions. Device settings 1 to 4 were evaluated. To allow accurate & repeatable comparison, we employed a bench breathing simulator (ASL5000, IngMar Medical, Pittsburgh, PA, USA) [8–10, 12, 19], with an inline low impedance flowmeter for pulse visualisation (PF-300 FlowAnalyser, IMT Medical, Buchs, Switzerland). Like others [8], we coupled the breathing simulator to the POC cannula via an anatomically realistic bench nose and an adjustable ‘oral’ breathing route. We used a custom ‘effort’ profile developed in-house due to the non-physiologic offerings on commercial breathing simulators. The shape of the ‘patient effort’ is a crucial determinant of the inspiratory flow amplitude and shape, and thus immensely influences device triggering, be it triggering of a ventilator breath or of an oxygen pulse.

Our in-house effort profile comprises:

- A second-order polynomial inspiratory profile based on the recommendations of Yamada et al. [20] and Milic-Emili et al .[21];
- A square-law post-inspiratory decay [22] with timing constraints;
- During periods of high ventilatory demand, an active expiratory contribution in the form of a skewed sinusoid is added as needed to partially defend against rampant hyperinflation [23]. Expiratory effort contribution is expressed as a percentage of peak inspiratory effort.

**Fig. 2:**
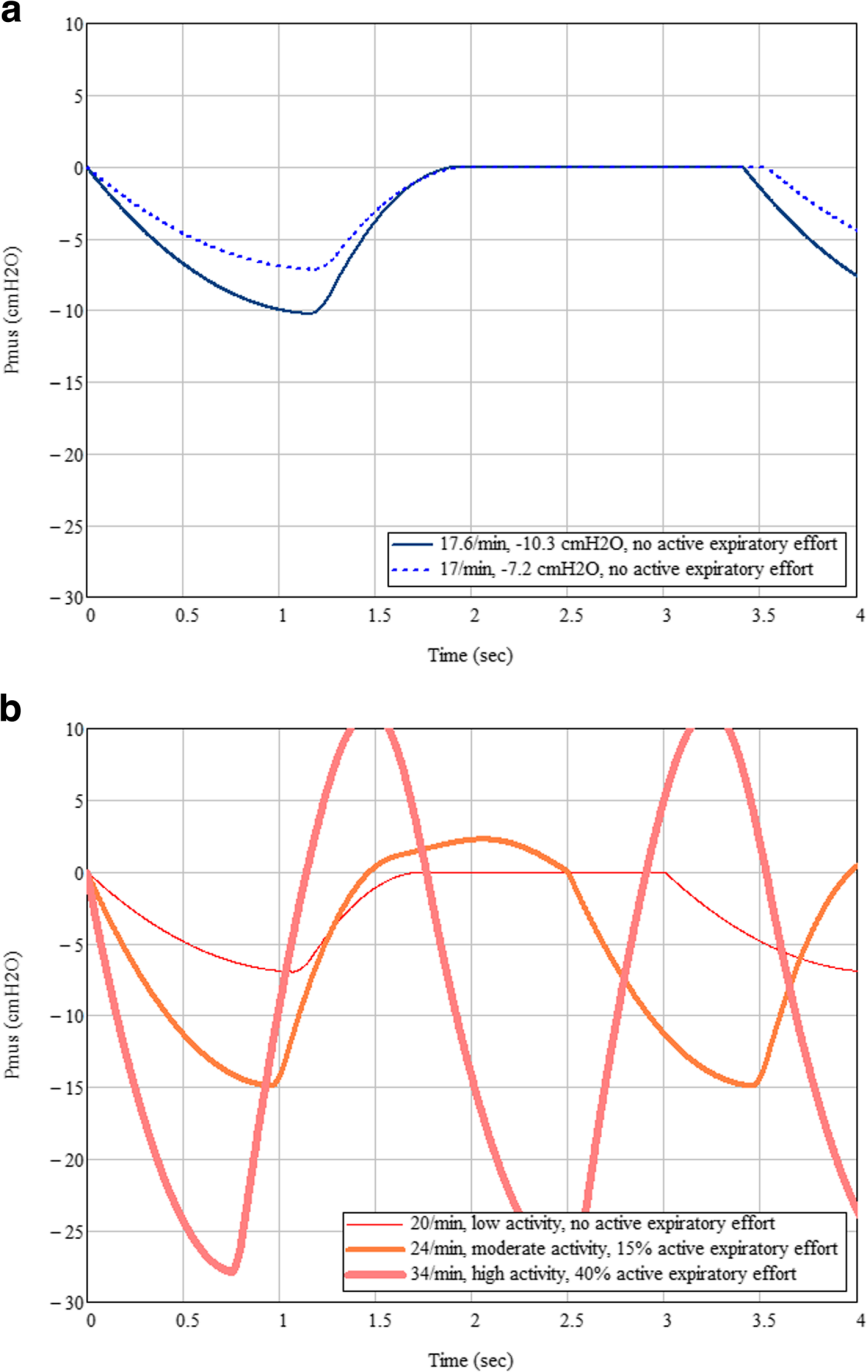
Examples of respiratory effort used for simulated breathing. **a** Effort profile used for resting awake breathing (dashed, − 7.2cmH_2_O amplitude, 17/min) and asleep breathing (solid, − 10.3 cmH_2_O amplitude, 17.6/min). **b** A sample of efforts used in vigorous breathing sequence, at baseline (thin, −7cmH_2_O amplitude, 20/min, no expiratory effort), moderate activity (medium thickness, −15cmH_2_O amplitude, 24/min, 15% expiratory effort), and high exertion (thick, −28cmH_2_O, 34/min, 40% expiratory effort)

Figure 2 offers examples of the respiratory effort waveform (further details may be made available on request).

**Fig. 3:**
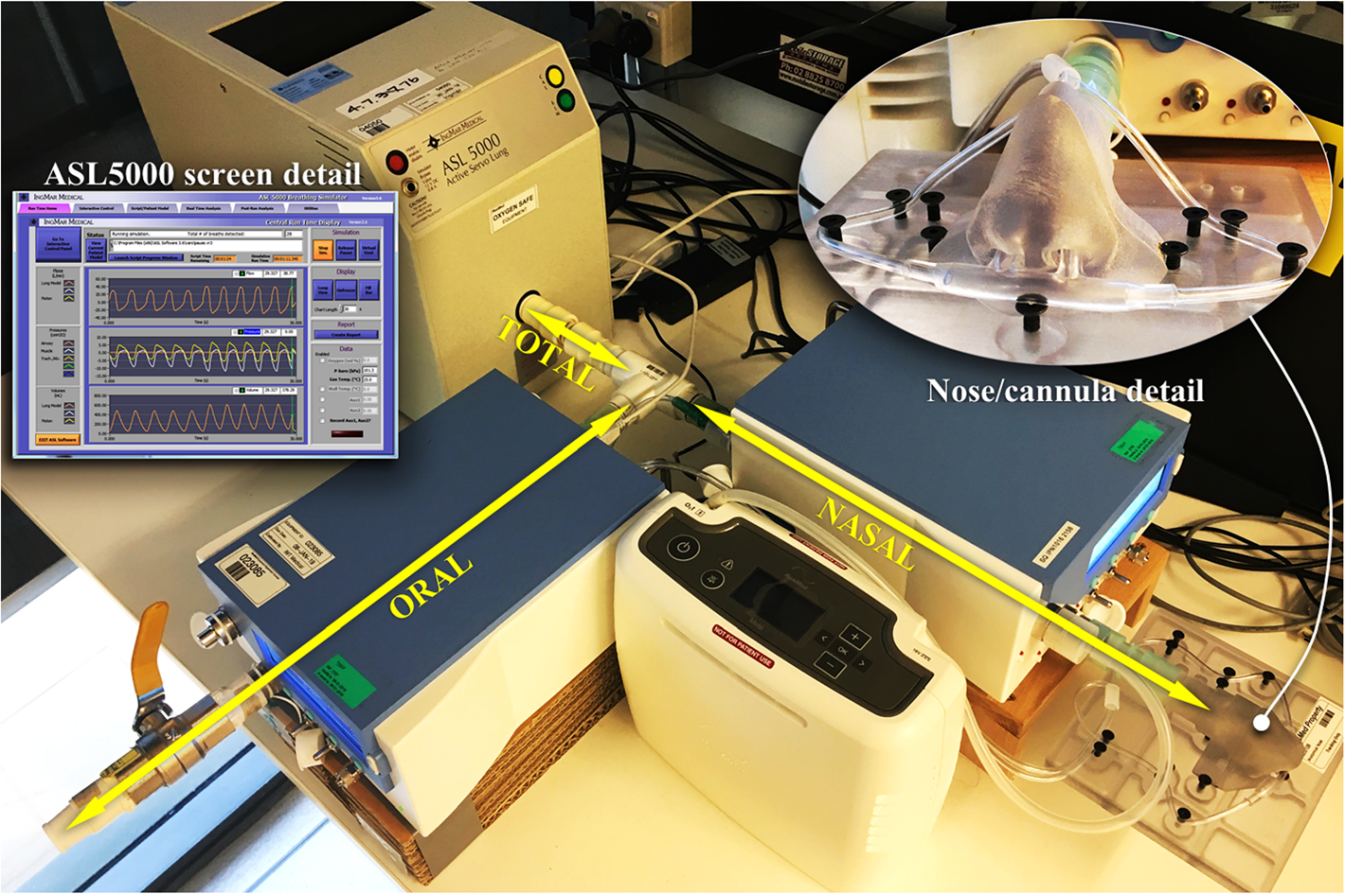
Bench setup, showing nose with cannula fitted, POC device, inline flowmeters and breathing simulator. Inset is an example of the breathing simulator user interface and a close-up of the nose/cannula

The nose model was 3D printed from MRI data of a Caucasian adult male of average height, age mid-30s; a photograph is shown in Fig. 3. The oral route consisted of a T-connector and adjustable valve. Flow through both breathing routes was metered, as was the delivered oxygen pulse (FlowAnalyser PF-300, IMT Analytics, Switzerland). During each test, oronasal partitioning remained fixed and the cannula was fully inserted and stable.

## Results

### Inspiratory synchronization with high ventilatory demand

**Fig. 4:**
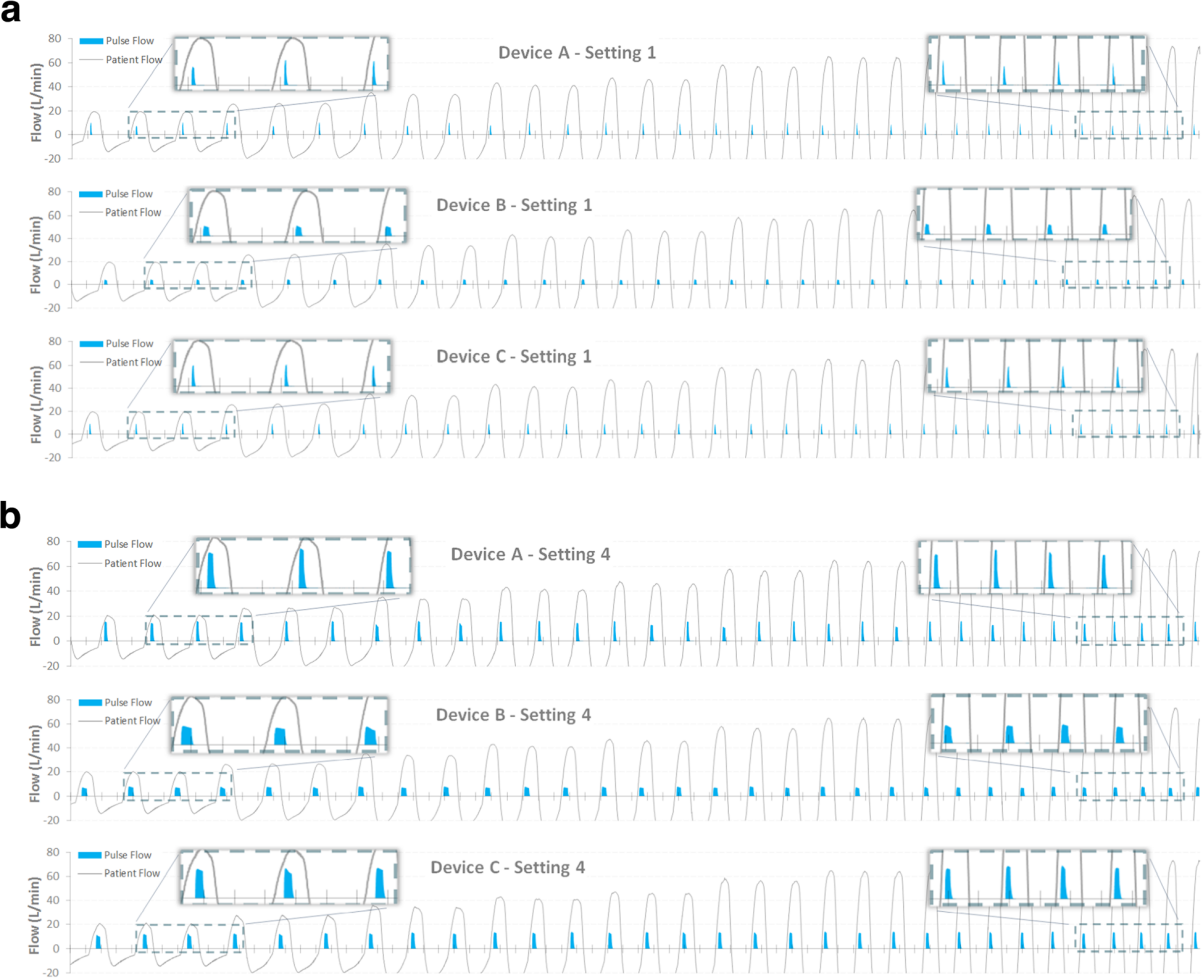
POC triggering performance at (**a**) POC setting 1 & (**b**) POC setting 4 for a simulated stable COPD patient during exercise, 100% nasal breathing

Figure 4 presents results of bench tests for the 3 representative POC devices during simulated vigorous activity and 100% nasal breathing. A compressed timescale illustrates the entire breath sequence. Pulse synchrony and alignment within every breath was good for all devices at all settings from 1 to 4, with no evidence of spurious triggers. For brevity only the data for settings 1 and setting 4 are shown.

### Inspiratory synchronization at rest

**Fig. 5:**
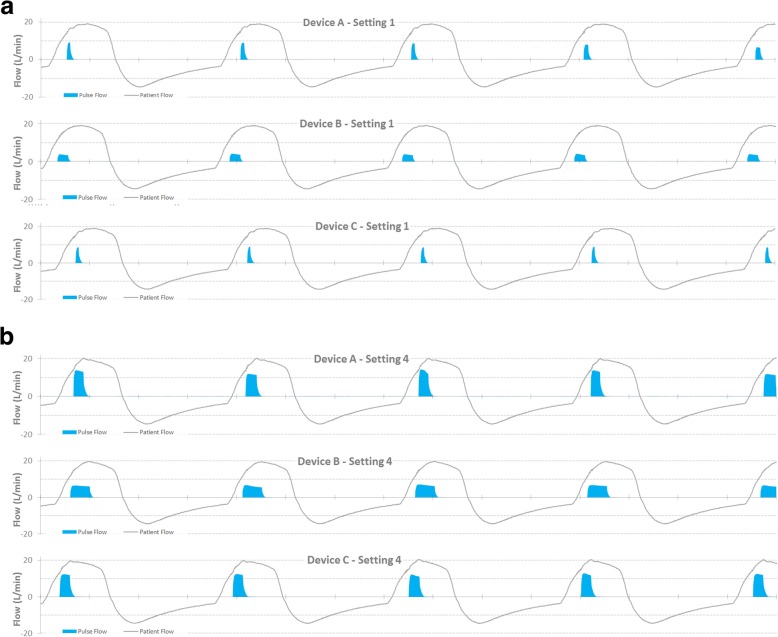
POC triggering for 3 POC devices treating a simulated small adult awake COPD patient at rest, 100% nasal breathing; (**a**) POC setting 1 and (**b**) POC setting 4

Figure 5 (a) and (b) show results of a simulated COPD patient with low ventilatory demand at rest and 100% nasal breathing. The traces are expanded to allow the alignment of the oxygen pulse with each breath to be viewed, for POC settings 1 and 4 respectively. All three POC devices perform well, with 100% triggering success & reasonable pulse alignment with the alveolar duration of the inspiration, and with no spurious triggering. Results for setting 2 & 3 are omitted for brevity.

### Inspiratory synchronization during oronasal breathing (nasal fraction reduced)

**Fig. 6:**
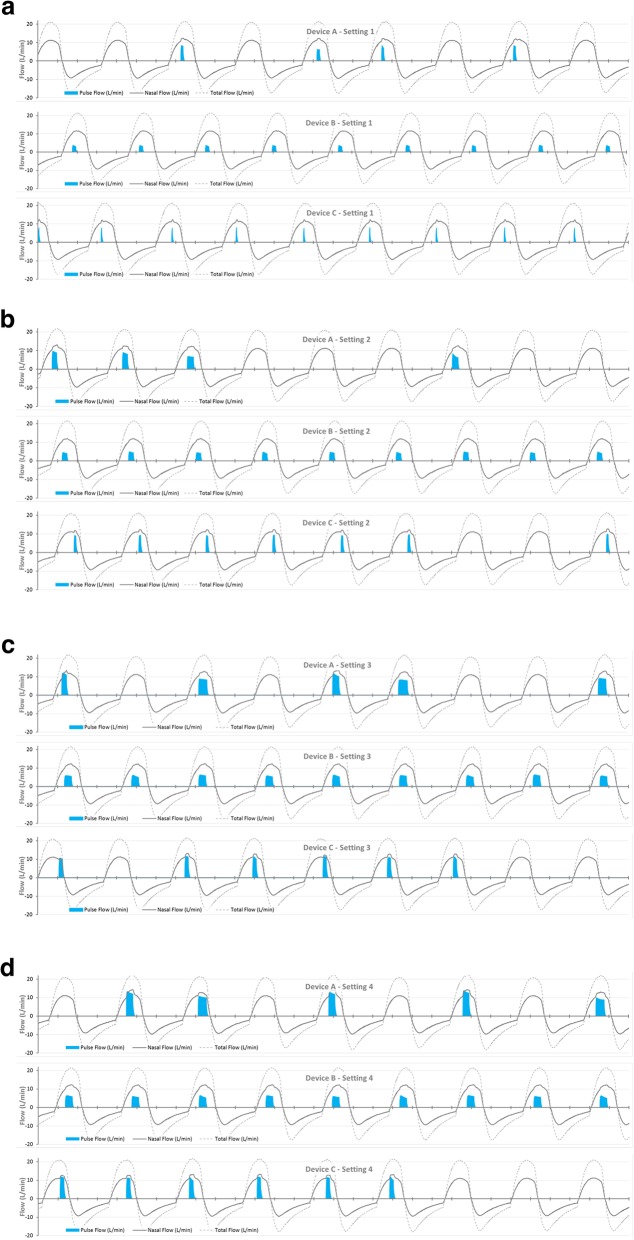
POC triggering for 3 POC devices treating a simulated sleeping adult COPD patient breathing oronasally (nasal 53%, 182 mL); (**a**) POC setting 1, (**b**) POC setting 2, (**c**) POC setting 3, and (d) POC setting 4

The third behaviour investigated was that of a small *nasal* tidal volume of 182 mL, representing 53% of the total (oronasal) inspiratory volume (343 mL). Representative synchrony performance for device settings 1, 2, 3 & 4 are charted in Fig. 6 (a) to (d) respectively. Table 2 compares the proportion of POC pulses aligned with inhalation, analysed across the final 78 consecutive breaths of the breathing sequence. Pulse synchrony with this breathing behaviour is more diverse, spanning from 40 to 100% depending on the POC device and the POC output setting.

**Table 2:** Trigger results for shallow nasal breathing, proportion of pulses aligned with inhalation

POC Setting	Proportion of POC pulses aligned with inspiration		
	Device A	Device B	Device C
1	42%	100%	99%
2	40%	100%	90%
3	42%	100%	51%
4	44%	100%	78%

## Discussion

The focus of this study was the ability of three contemporary POC devices to detect inhalation and deliver a corresponding pulse, a fundamental objective of pulsed oxygen delivery.

Portable oxygen concentrators are necessarily limited in their oxygen production and battery reserve, hence efficient use of oxygen is paramount. Any portion of the pulse which does not reach the user’s alveoli may represent waste. A POC’s output setting may be increased to compensate for such wastage, but then the battery operating duration will suffer. So regardless of a device’s oxygen production capacity^1^ or dosing scheme, the correct alignment of the pulse with inhalation can be critical.

Inspiratory synchrony is the alignment of the pulse start and pulse finish relative to the user’s inspiratory flow. An example of pulse alignment within a breath can be seen in Fig. 1.

Note that inspiratory synchrony is just one of numerous elements of pulse delivery that may affect efficacy. As seen in Fig. 1, it is the *area* of the pulse waveform reaching the alveoli that determines the functional oxygen volume delivered per breath, dictated by factors such as pulse amplitude, pulse duration and how the oxygen output is rationed as breath rate changes. These important issues are not the subjects of this triggering study, beyond noting that trigger timing can also have implications for these issues.

### Inspiratory synchrony -- pulse termination

If we are to avoid wasting oxygen in the anatomic dead space (dark shaded zone in Fig. 1) the pulse must be fully delivered within the alveolar portion of the breath, irrespective of pulse volume. For a normal subject at rest, the anatomic dead space represents about one third of the tidal volume and the ‘alveolar’ duration represents about the first 60% of the inspiratory duration. If a subject’s breathing becomes shallower than typical, multiple factors can affect pulsed oxygen efficacy: (a) triggering may be delayed due to the weaker inspiratory flow, (b) tidal volume is reduced but the anatomic deadspace is not, hence the ‘alveolar’ duration is shorter, and (c) if the oxygen pulse flow exceeds inspiratory flow, oxygen may be wasted due to pooling. Issues (a) and (b) both contribute to late pulse termination and associated wastage, and both may be countered by triggering the pulse early within inspiration.

### Inspiratory synchrony -- pulse initiation (triggering)

Delivering the pulse early within inspiration is facilitated by sensitive and responsive triggering. But care is needed to avoid introducing a problem: false triggering. False triggering not only wastes oxygen, but risks loss of synchrony on subsequent breaths. So the objectives for a trigger should consider both sensitivity and robustness, such as:

- Compatible with a wide range of users, large and small.
- Maintain synchrony with the user *across a wide range of behaviours*, from sleep to rest to vigorous activity.
- Minimal spurious triggering.
- Be as early as possible within the above constraints.

### Inspiratory synchrony performance during exertion and rest

Our bench testing of these three contemporary POCs during vigorous breathing (Fig. 4) and at rest (Fig. 5) revealed all devices showed excellent pulse alignment at all POC settings. Each breath is rewarded with a pulse, and the pulse terminates approximately within the first 60% of the start of the breath.

The exertion scenario confirms these devices successfully track dynamically changing breath rates up to the highest rate simulated (34/min), albeit with the proviso of 100% nasal breathing.

### Inspiratory synchrony performance during oronasal breathing (nasal fraction reduced)

Figure 6 shows the varying ability of these POC devices to synchronize with a shallow *nasal* inspiratory volume of 182 mL (breath rate 17.6/min), 53% of the total breath volume. Two of the three devices did not achieve full synchrony at all settings.

This test offers far greater trigger challenge than typical for POC bench evaluations, so discussion is warranted. First, the scenario depicted is that of sleep, where the efficacy and appropriateness of pulsed oxygen devices has been questioned [2, 19, 24, 25]. Yet the efficacy of the ‘reference’ nocturnal oxygen therapy, *continuous* flow oxygen via nasal cannula, also shows high variability during sleep [26]. Sleep introduces issues with potential to influence *any* oxygen therapy delivered via nasal cannula. In normal subjects, sleep is associated with reduced ventilation than when awake, at a similar or slightly increased breath rate, hence the breaths are 6–25% shallower depending on sleep stage [27]. Similar behaviour is observed in COPD and other nocturnal desaturators, but sometimes with *profound* reduction in tidal volume in REM sleep [15, 28]. Other identified sleep issues include: a worsening of gas exchange ability; ‘mouth breathing’; a displaced cannula; and other sleep breathing disorders (snoring, obstructive apnoea, periodic breathing) [24, 26, 29, 30]. For such issues sensitive triggering may promote delivery of the pulse within the alveolar duration, or may dictate whether inhalation is detected at all. Continuous flow oxygen *may* be less vulnerable to sleep issues, given its delivery is unaffected by breathing behaviours and it offers the (situational) possibility of oxygen pooling. But as noted by Chatburn et al. [24], a key consideration in achieving efficacious therapy of any oxygen therapy “is not *whether* a person desaturates at night, but *why* they desaturate”. And despite the controversy, the ambition for the POC category is evolving towards a single-device for home and ambulation, as experience grows with nocturnal pulsed oxygen delivery and in response to user preference [24, 31, 32].

Second, the test scenario depicts substantial oronasal breathing, where the POC does not ‘see’ the full inhaled volume. Clearly 100% mouth breathing for sustained periods will confound any style of nasal cannula oxygen therapy, hence ‘mouth breathing’ is a commonly cited concern for nasal oxygen therapy during sleep. But from the limited research data available, *exclusive* mouth breathing during sleep is infrequent: in one early study in healthy sleeping subjects, 100% mouth breathing was not seen at all [17], while other sources suggest this may occur in less than 5–10% of normal subjects [33, 34]. But ventilation shared between nose and mouth during sleep – the scenario represented in Fig. 6 – is frequently seen, particularly in men and increasingly with age [35]. Our scenario depicted a nasal fraction of 53%, aligned with values seen in older subjects within a study [17] on mouth breathing in a sleeping normal cohort.

Third, our test case investigating oronasal breathing may be instructive for *daytime* situations where oronasal breathing may diminish efficacy of nasal oxygen. In awake healthy subjects, dominant mouth breathing at rest and during exercise is quite rare (5% of subjects), with little apparent increase with age [36]. But in a population with respiratory compromise, Chadha et al. [37] found a nasal ventilation fraction at rest (awake) of around 56% compared to 86% for healthy subjects. Leiberman et al. [38] found that during exertion the nasal fraction decreased in all subjects, but more so in those with respiratory compromise (nasal fraction reduced to 25%). Based on these limited data, it seems oronasal breathing may be common, but it is unusual for the nasal fraction to drop to zero for sustained periods. So it may be that if a POC’s trigger were sufficiently sensitive, it may remain efficacious across the majority of oronasal or ‘mouth breathing’ instances.

Fourth, the interface between cannula and nose possesses ‘geometric’ factors that may affect the capability of the POC to detect inhalation. Consider the nature of the POC trigger: inspiratory flow induces a reduction in pressure at the cannula tip; this pressure is sensed and if below a threshold value, a trigger is asserted. The change in pressure induced at the cannula tip depends not only on the magnitude of nasal flow, but also on various geometric factors, including:

- Nare internal geometry, itself a function of individual differences, age, race.
- Nasal valve geometry (depth, area, shape).
- Cannula tip geometry
- Cannula insertion depth into the nare, and position relative to nasal valve.

The net effect of these listed factors may result in wide variability of trigger performance between individuals, despite a similar ventilation pattern. This has been evaluated on the bench using replica adult airways [8, 39]. Across the different replicas, researchers found more than 3-fold variation in the amount of pressure developed for a given nasal flow. A commercial POC included in their investigation proved *unable to trigger* on three of the 15 replicas when used with their sleep breathing pattern (520 mL tidal volume) at setting 2 [8].

Finally, almost all children receiving long term oxygen therapy also require ambulatory oxygen therapy [40]. Pulsed oxygen delivery is generally considered inappropriate for babies & very small children, but for larger children sensitive triggering may determine whether a child can enjoy the ambulatory benefits of pulsed oxygen delivery.

Overall, a more sensitive trigger may translate to greater likelihood of success across high inter-subject variability in oronasal breathing and in anatomic variation, and across diverse behaviours within a patient. But high sensitivity must not come at the expense of spurious triggering, which can dramatically impair pulsed oxygen efficacy. In this assessment, the most sensitive of the devices tested did not display inadvertent triggering across any of the simulated behaviours.

There are limitations to the bench research presented here. The scope was limited only to the POC’s ability to detect inspiration and trigger a pulse, with no consideration of other pulse parameters such as the pulse’s amplitude, pulse volume, or how much of that volume was successfully delivered within the ‘alveolar’ duration. The tests were conducted in a controlled static laboratory environment free of drafts and ambient vibration. It employed a single bench ‘nose’ with stable cannula positioning. These simplifications allowed us to focus on repeatable and accurate comparison of device triggering, but lack the complexities of real patient breathing and ambient effects, and the results may not relate directly to efficacy of oxygenation.

## Conclusion

Portable oxygen concentrators are expanding in popularity, and may have potential to act as a single oxygen therapy device (as opposed to a stationary system and an ambulatory system). Success as a single device will depend on the confidence that pulsed oxygen delivery is efficacious across the breadth of patient breathing behaviours. These behaviours may span from quiet breathing (during sleep and at rest) through to vigorous activity, and a variety of oronasal breath partitioning across these activities. A wide variety of nasal geometries also exist which can influence the ability to detect inspiratory flow, as can sub-optimal positioning of the cannula. Such factors can affect the efficacy of pulsed oxygen delivery in an individual user and across users, and suggest there may be clinical benefit in a sensitive yet robust trigger. In this study, all devices performed well with the simulated COPD patient at rest and at elevated breath rates. Performance diverged during oronasal breathing due to differences in trigger sensitivity. Sensitive triggering may offer practical advantage in various scenarios, given the diversity in factors such as patient size, nasal geometry, nocturnal breathing, and the partitioning of ventilation between nose and mouth across patient activity. Factors such as these may contribute to the variability in efficacy observed across pulse oxygen devices.

## Data Availability

The author confirms that all pertinent data are represented graphically within the article. Further details on methods may be made available on reasonable request.

## Abbreviations

/min: Per minute
3D: Three-dimensional
cmH_2_O: centimetres of water (unit of pressure)
COPD: Chronic Obstructive Pulmonary Disease
kg: kilogram
L/min: Litres per minute
L/sec: Litres per second
LOX: Liquid Oxygen
mL: millilitres
MRI: Magnetic resonance imaging
POC: Portable Oxygen Concentrator
PODS: Pulsed Oxygen Delivery System
REM: Rapid eye movement

## Footnotes

1 In Leblanc’s study [7] , saturation achieved at maximum output of a 1 L/min device (4.6 kg) was similar to of a 3 L/min device (8.6 kg).
